# Seasonal Variation in Conjunctivitis in Saudi Arabia and Reduction of Searches During the COVID-19 Pandemic: Analysis of Google Trends Data

**DOI:** 10.2196/73845

**Published:** 2025-07-21

**Authors:** Abdulaziz S AlHarthi

**Affiliations:** 1 Ophthalmology Department, College of Medicine, Majmaah University, Al-Majmaah, 11952, Saudi Arabia, +966557331130

**Keywords:** Google Trends, conjunctivitis, COVID-19, seasonality

## Abstract

Analysis of Google Trends data for the Arabic term for “conjunctivitis” found significant seasonality, with the highest interest in February and the lowest in August (*P*<.001); moreover, during COVID-19 restrictions, relative search volume decreased by 53.3%, with a disruption of the seasonal pattern.

## Introduction

Conjunctivitis is one of the most common eye-related infections worldwide, and it frequently results in nonemergency ophthalmic visits to health care facilities [1]. Previous studies have observed seasonal patterns in the incidence of cases. In the United States, cases have been reported to be predominant in the winter and spring [2].

During the COVID-19 pandemic, preventive measures to decrease transmission were implemented worldwide. In the Kingdom of Saudi Arabia, this included the obligation to wear a face mask, social distancing, and the suspension of gatherings in educational institutions. A curfew was imposed in late March 2020; all measures were lifted entirely in March 2022.

To date, few studies have explored the impact of COVID-19 restrictions on the spread of other infectious diseases. Here, we explore Google Trends data to determine whether public search volumes for conjunctivitis in Saudi Arabia exhibit seasonality and how this was impacted during the COVID-19 period.

## Methods

### Overview

Monthly normalized relative search volume data (0‐100) were extracted as CSV files from Google Trends in September 2024 for the search term “الرمد” (Arabic for “conjunctivitis”), with the location set to Saudi Arabia and the period from January 2011 to October 2024, without a category selection. Statistical analyses were conducted in R (version 4.4.2; R Foundation) with the *Forecast*, *seatest*, and *season* packages.

Poisson cosinor analysis was performed to assess seasonality during the pre–COVID-19 period (2011‐2019), avoiding pattern distortions due to COVID-19–related search behavior, with the significance level set at .025, consistent with previous studies [3]. To estimate the impact of COVID-19 restrictions, a log-transformed autoregressive integrated moving average (ARIMA) model (1,0,0) was fitted on data from 2011 to 2023, including binary variables for the intervention period (March 2020 to March 2022), monthly dummy variables, lag-12, and linear and quadratic trends (significance at *P*<.05). Residual diagnostics indicated approximate normality and passed the Ljung-Box test and passed the Ljung-Box test (χ^2^=16.19; *P*=.06) which indicates white noise in the residuals. Seasonality disruption was assessed using the Friedman test across the years before and during COVID-19.

### Ethical Considerations

The data are public source, so no institutional review board approval was needed.

## Results

**Figure 1.**
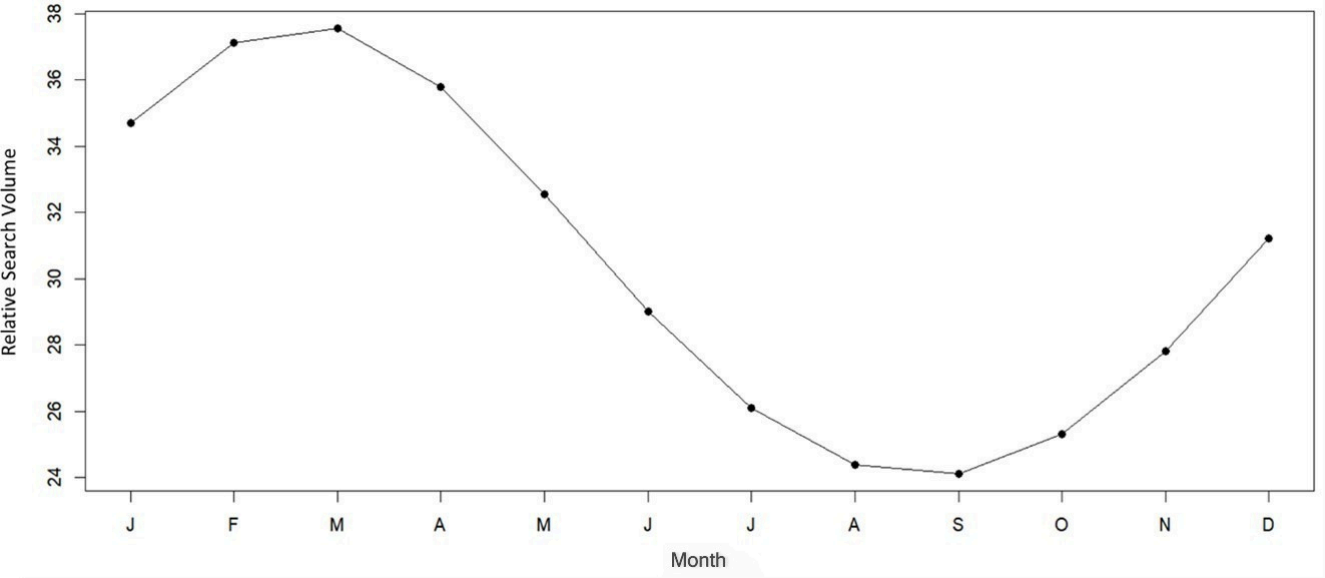
Cosinor model plot for conjunctivitis seasonality in Google Trends search volume in Saudi Arabia for the Arabic word for “conjunctivitis” for the period from January 2011 to December 2019.

Cosinor analysis confirmed a statistically significant seasonal pattern in the relative search volume for conjunctivitis (amplitude=7.57, phase month=2.7, and low-point month=8.7; *P*<.001; Figure 1). A relative search peak was observed in February and the lowest volume during summer, in August. An interrupted model using ARIMA showed an estimated 53.3% reduction (95% CI −43.2% to 61.6%; *P*<.001) in the relative search volume for conjunctivitis during the COVID-19 quarantine compared to the pre–COVID-19 period. Notably, the Friedman test showed that the prepandemic seasonal pattern (when the value was *P*=.03) was disrupted during pandemic restriction policies from 2020 to 2022 (when the value changed to *P*=.20).

## Discussion

### Principal Findings

This study shows that there is significant seasonality in the search volume for conjunctivitis. Moreover, there was a significant reduction in and disruption of seasonality during the pandemic, providing a line of evidence to complement traditional methods; the finding suggests that the preventive strategies taken to lower the risk of COVID-19 transmission may have also reduced the incidence of conjunctivitis or at least online search interest.

Previous studies from the United States have explored the seasonality of searches for conjunctivitis and obtained similar findings, that is, that search increases from fall to spring [2]. Several factors lead to an increased risk of conjunctivitis during winter, including indoor crowdedness and an increased risk of contact with infected people, especially in schools. Additionally, conjunctivitis cases increase with other infections, like the flu, that exhibit seasonal variation.

Adenoviral conjunctivitis has a similar transmission mechanism as coronaviruses [4], with transmission rates of 30% in nursing homes and 76% in schools [5,6]. Recently, Deiner et al [7] showed that there was a sustained reduction in searches for communicable diseases, including conjunctivitis, during the pandemic in the United States. In a study from Spain, there was a decrease in the incidence of conjunctivitis by 48.5% during the pandemic in 2020 [8]. Additionally, Lavista Ferres et al [9] published evidence of a decrease in nonallergic conjunctivitis cases in the emergency department of 37.3%.

### Limitations

These findings are based on Google Trends search volume, which may be influenced by other factors, such as the media, not only by the actual incidence of conjunctivitis. Therefore, further studies to elaborate on the seasonality and impact of COVID-19 restrictions are needed.

### Conclusions

These findings highlight the potential seasonality of conjunctivitis. Understanding disease patterns and the role of public health measures against COVID-19 in reducing conjunctivitis spread has public health implications for the reduction of conjunctivitis prevalence.

## Abbreviations

ARIMA: autoregressive integrated moving average
